# Supplementary material for: PSTPIP1-Associated Myeloid-Related Proteinemia Inflammatory (PAMI) Syndrome: A Systematic Review
Source: Genes (Basel). 2023 Aug 19;14(8):1655. doi: 10.3390/genes14081655 (PMC10454568; doi:10.3390/genes14081655)
Supplement: Supplementary file 1 [file genes-14-01655-s001.zip › File S1 Search strategies.docx]

**Search strategies**

Medline ALL Ovid 1946 to October 12, 2022

58 references 13.10.2023

((PSTPIP1* OR "proline-serine-threonine phosphatase-interacting protein 1") ADJ12 (inflammatory OR autoinflammatory)).ab,ti,kf OR (PAMI ADJ6 syndrome).ab,ti,kf OR ((Hyperzincaemia* OR Hyperzincemia*) AND (hypercalprotectinaemia* OR hypercalprotectinemia*)).ab,ti,kf OR ((PSTPIP1* OR "proline-serine-threonine phosphatase-interacting protein 1") AND (e250k OR e257k)).ab,ti,kf

Embase.com

124 references 13.10.2023

((PSTPIP1* OR "proline-serine-threonine phosphatase-interacting protein 1") NEAR/12 (inflammatory OR autoinflammatory)):ab,ti,kw,de OR (PAMI NEAR/6 syndrome):ab,ti,kw,de OR ((Hyperzincaemia* OR Hyperzincemia*) AND (hypercalprotectinaemia* OR hypercalprotectinemia*)):ab,ti,kw,de OR (('pstpip1 gene'/de OR (PSTPIP1* OR "proline-serine-threonine phosphatase-interacting protein 1"):ab,ti,kw) AND (e250k OR e257k):ab,ti,kw)

Web of Science – Core collection

70 references 13.10.2023

TS=(((PSTPIP1* OR "proline-serine-threonine phosphatase-interacting protein 1") NEAR/12 (inflammatory OR autoinflammatory)) OR (PAMI NEAR/6 syndrome) OR ((Hyperzincaemia* OR Hyperzincemia*) AND (hypercalprotectinaemia* OR hypercalprotectinemia*)) OR ((pstpip1* OR "proline-serine-threonine phosphatase-interacting protein 1") AND (e250k OR e257k)))
