# Supplementary material for: PSTPIP1-Associated Myeloid-Related Proteinemia Inflammatory (PAMI) Syndrome: A Systematic Review
Source: Genes (Basel). 2023 Aug 19;14(8):1655. doi: 10.3390/genes14081655 (PMC10454568; doi:10.3390/genes14081655)
Supplement: Supplementary file 1 [file genes-14-01655-s001.zip › File S2 PAMI Review patient description .docx]

Case presentation

We present the case of a 13-month-old Caucasian female patient referred to our pediatric rheumatology unit in 2018 due to recurrent episodes of osteoarthritis. The patient was born to non-consanguineous healthy parents with unremarkable personal and family history. At 5 months, she developed fever and leg pain, which led to the diagnosis of infectious osteoarthritis. This had a slow resolution when treated with ceftriaxone. However, a second episode of osteoarthritis occurred at 12 months of age, and the bacterial cultures from blood and synovial fluid were negative, which made an infectious etiology unlikely.

Once uncommon infections, metabolic disorders, and malignancies had been ruled out, the patient was referred to our center.

At that time, the patient displayed failure to thrive, hepatosplenomegaly, and painful limitation in active and passive movements of the right shoulder. She had a normal motor development.

Blood tests revealed systemic inflammation, anemia, neutropenia and thrombocytosis. Detailed list of laboratory findings is shown in table 1.

Bone marrow aspiration and biopsy revealed an increased trilinear cellularity, with left deviation and signs of megakaryocyte lineage, predominance (and to a lesser degree level of the erythroid lineage). No excess of blasts and no fibrosis. Morphological and immunophenotypic analysis did not show any arguments for lymphoproliferative B or T syndrome or excess of large granular lymphocytes or NK lymphocytes.

Immunologic investigations including serum immunoglobulin, C3, C4 levels, and lymphocyte phenotyping were normal. Levels of four cytokines (TNFα, IL-6, IL-8, IL -1 Ra) were measured. The result showed a remarkable increased level of IL-18 and IL-1 Ra.

**Table 1. Abnormal Blood Investigations in our patient at diagnosis**


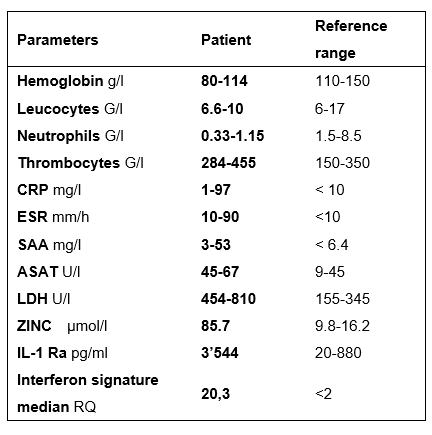


Due to the patient history of recurrent osteoarthritis, we performed a whole-body MRI that confirmed the presence of multifocal osteomyelitis.

At 15 months, Ibuprofen (40mg/kg/day) was initiated with iron substitution. Treatment led to the disappearance of osteo-articular manifestations and biologic inflammation around three months after the start of therapy (Figure2). The patient’s anemia improved but hepatosplenomegaly and neutropenia remained unchanged.

Because of the early onset multifocal osteomyelitis and the high level of IL-1Ra, a genetic disorder was suspected and whole exome sequencing was performed. An heterozygous G>A transition at c.748 position in exon 11 of PSTPIP1 gene leading to the missense p.E250K variant was found. Ultimately, based on patient’s medical history, clinical parameters and genetic result, the diagnosis of PSTPIP1-associated myeloid-related proteinemia inflammatory (PAMI) syndrome was made.

To note that the same missense was found in the father who was clinically and biologically asymptomatic (CRP, ESR, SAA and BCT were normal). Consistently with the reported literature, blood zinc level was significantly elevated at 85.7 µmol/l [9.8-16.2 µmol/l]. Unfortunately, MRP 8/14 was not available for testing.

During a follow-up period of 5 years, our patient exhibited significant improvement while receiving ibuprofen treatment, resulting in the resolution of osteoarticular manifestation and biologic inflammation. Throughout this period, no new symptoms related to PAMI syndrome were observed. Neutropenia, anemia, and hepatosplenomegaly remained unchanged. However, she experienced recurrent acute otitis media and one episode of cutaneous abscess caused by Streptococcus spp.

The ibuprofen treatment was maintained for a duration of 2 years and subsequently discontinued. Unfortunately, after 18-month period, our patient suffered from relapse characterized by a flare of osteomyelitis and systemic inflammation. However, she demonstrated a favorable response upon resuming ibuprofen. Give the patient’s dependance on NSAIDs, there is an ongoing discussion regarding the possibility of initiating treatment with canakinumab to control the systemic inflammation and arthralgia.
