# Supplementary material for: PSTPIP1-Associated Myeloid-Related Proteinemia Inflammatory (PAMI) Syndrome: A Systematic Review
Source: Genes (Basel). 2023 Aug 19;14(8):1655. doi: 10.3390/genes14081655 (PMC10454568; doi:10.3390/genes14081655)
Supplement: Supplementary file 1 [file genes-14-01655-s001.zip › PAMI review Abbreviations.docx]

PSTPIP1: Proline-serine-threonine phosphatase interacting protein 1

PAMI: PSTPIP1-associated myeloid-related proteinemia inflammatory

*PAID: PSTPIP1*-associated inflammatory diseases

PAPA: pyogenic arthritis, pyoderma gangrenosum, acne

MRP: myeloid-related protein

PRISMA: Preferred Reporting Items for Systematic reviews and Meta-Analyses

TNF: HSCT:
NSAIDs : non-steroidal anti-inflammatory drugs

cDMARDs: conventional disease-modifying anti rheumatic drugs

bDMARDs: biological disease-modifying anti rheumatic drugs

WBC: white blood count

ANC: absolute neutrophil count

CRP: C-reactive protein

SAA: serum Amyloid A

ESR: erythrocyte sedimentation rate

Hb: hemoglobin

HTAP : pulmonary arterial hypertension

MAS : Macrophage activation syndrome

IL-1 : Interleukin-1

IL-6 : Interleukin-6

G-CSF: granulocyte colony-stimulating-factor
